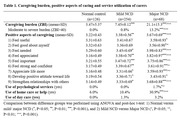# Caregiving experience and service utilization of informal caregivers for community‐living older adults with neurocognitive disorders: preliminary findings in Hong Kong

**DOI:** 10.1002/alz.089312

**Published:** 2025-01-09

**Authors:** Zhaohua Huo, Allen TC Lee, Benjamin HK Yip, Cuichan Lin, Suk Ling Ma, Samuel YS Wong, Linda CW Lam

**Affiliations:** ^1^ The Chinese University of Hong Kong, Hong Kong China; ^2^ The Chinese University of Hong Kong, Hong Kong, Hong Kong Hong Kong

## Abstract

**Background:**

Caring for people with dementia in communities inevitably brings heavy burden for their family caregivers. Senses of positive aspects from caring and proper service support may help shape a healthy care journey for carers. This study aims to evaluate the caregiving burden, positive aspects, and service utilization of informal caregivers for community‐living older adults with neurocognitive disorders (NCDs).

**Method:**

448 informal caregivers of older adults aged ≥60 (major NCD/ dementia: 68, mild NCD: 254, normal cognition: 126) were recruited from in a community‐based prevalence survey in Hong Kong. Caregivers’ subjective burden was measured by Zarit Burden Interview (ZBI), while their gain was measured by positive aspect of caregiving scale (PACS). Caregivers’ use of medical and social service was also recorded. Differences in measures across three disease groups were examined by analysis of variance and t‐test. Correlations between measures were estimated by Pearson coefficient.

**Result:**

Nearly 60% of interviewed carers were female, spouses of care recipients, and not in employment. Over 75% of them cohabited with care recipients and take the major responsibility for caring. Compared to normal controls, carers of major and mild NCD had higher scores in caring burden, and over one in ten dementia carers were suffering moderate to severe degree of burden. Regarding positive aspects, carers of major and mild NCDs also have higher scores, mainly in items of “feel needed”, “feel appreciated”, and “feel important”. As for service utilization, dementia carers used more psychological and home help services. Finally, caring burden was positively correlated to positive gain (r = 0.233, P<0.001), psychological (r = 0.186, P<0.001) and social services (r = 0.146, P<0.001). The sense of positive aspects was uncorrelated to psychological services (r = ‐0.064, P = 0.174) but positively correlated to social services (r = 0.149, P = 0.002).

**Conclusion:**

Informal caregivers of major and mild NCD are experiencing heavier caring burden. They also have higher sense of positive aspects from caring and more utilization of psychological and social care. Relationships between positive and negative experience of caregivers and their service utilization need further investigations.